# A human monoclonal antibody against HPV16 recognizes an immunodominant and neutralizing epitope partially overlapping with that of H16.V5

**DOI:** 10.1038/srep19042

**Published:** 2016-01-11

**Authors:** Lin Xia, Yangfei Xian, Daning Wang, Yuanzhi Chen, Xiaofen Huang, Xingjian Bi, Hai Yu, Zheng Fu, Xinlin Liu, Shaowei Li, Zhiqiang An, Wenxin Luo, Qinjian Zhao, Ningshao Xia

**Affiliations:** 1 State Key Laboratory of Molecular Vaccinology and Molecular Diagnostics, National Institute of Diagnostics and Vaccine Development in Infectious Diseases, School of Life Science, Xiamen University; Xiamen 361105, China; 2 Texas Therapeutics Institute, The Brown Foundation of Molecular Medicine, University of Texas Health Science Center at Houston, Houston TX77030, USA

## Abstract

The presence of neutralizing epitopes in human papillomavirus (HPV) L1 virus-like particles (VLPs) is the structural basis of prophylactic vaccines. An anti-HPV16 neutralizing monoclonal antibody (N-mAb) 26D1 was isolated from a memory B cell of a human vaccinee. The pre-binding of heparan sulfate to VLPs inhibited the binding of both N-mAbs to the antigen, indicating that the epitopes are critical for viral cell attachment/entry. Hybrid VLP binding with surface loop swapping between types indicated the essential roles of the DE and FG loops for both 26D1 (DEa in particular) and H16.V5 binding. Specifically, Tyr^135^ and Val^141^ on the DEa loop were shown to be critical residues for 26D1 binding via site-directed mutagenesis. Partially overlap between the epitopes between 26D1 and H16.V5 was shown using pairwise epitope mapping, and their binding difference is demonstrated to be predominantly in DE loop region. In addition, 26D1 epitope is immunodominant epitope recognized by both antibodies elicited by the authentic virus from infected individuals and polyclonal antibodies from vaccinees. Overall, a partially overlapping but distinct neutralizing epitope from that of H16.V5 was identified using a human N-mAb, shedding lights to the antibody arrays as part of human immune response to vaccination and infection.

Human papilloma virus (HPV) is the leading cause of cervical cancer, which is the second most common cancer in women worldwide^1^,^2^. Among the HPV oncogenic types associated with this carcinoma, HPV16 is the most prevalent and is responsible for approximately 70% of tumor specimens^3^,^4^. Infectious HPV is primarily composed of 72 pentamers (capsomeres) of the L1 protein, in association with up to 72 copies of L2 capsid protein^5^. The L1 pentamers have the intrinsic capacity to assemble into empty capsid-like structures referred to as papilloma virus-like particles (VLPs), with immunogenicity similar to infectious virions^6^,^7^,^8^. VLPs have been shown to develop high serum titers of neutralizing antibodies without substantial adverse effects^9^,^10^. VLPs can also be useful reagents for studies of viral receptor binding, entry mechanism, and capsid structure^11^,^12^. More importantly, VLPs have been used for the induction of protective immunity in animal models^13^,^14^,^15^ and the development of prophylactic vaccines for HPV infection^9^,^16^. In addition, passive transfer experiments have provided strong evidence that a neutralizing antibody response is sufficient to protect against HPV challenge^13^,^17^.

Each pentameric capsomer is composed of five monomers, with each monomer having five antigenic loops (BC, DE, EF, FG, and HI). Major neutralizing epitopes were found to be conformational sites that are located on these hypervariable loops^18^,^19^,^20^ and were recognized by type-specific neutralizing antibodies^21^,^22^. H16.V5 is a well-characterized HPV16 murine neutralizing antibody, and its binding to HPV16 VLPs completely blocks the reactivity of more than 75% of human antisera^23^. H16.V5 is often used for the assessment of the integrity and antigenicity of VLPs in vaccine products^24^. The antibody is known to recognize the FG and HI loops, which are immunodominant in the humoral response against the HPV16 major capsid protein^23^,^25^. A recent study mapped the precise conformational epitope of H16.V5 to 17 residues across five loops from two neighboring L1 proteins^12^. Moreover, half of the 17 binding residues targeted by H16.V5 were located in the FG loop, which supports the previous conclusion that residues at both ends of the FG loop are critical for H16.V5 binding^12^,^26^.

Current HPV vaccine strategies focus on generating not only type-specific antibodies but also cross-type neutralizing antibodies for broader type coverage^4^,^27^,^28^. Hybrid VLPs can be constructed in which particle assembly properties are retained with different loop grafting. Surface loops from two different HPV types can be grafted onto a single hybrid VLP to trigger an antibody response that be neutralizing to two different HPV types^27^,^28^. Therefore, identification of immunodominant regions of L1 protein is critical for designing cross-reactive hybrid VLPs. For antigenic determinants of HPV16 L1 VLP, because the reconstituted epitopes of HPV16 VLPs are generally identified by murine monoclonal antibodies such as HPV16.V5, epitope mapping on HPV16 virus has been hampered by limited antibody sources and a lack of structural information of the antibody-antigen complexes. For example, whether a human neutralizing antibody can recognize the same surface regions of H16.V5 has yet to be demonstrated.

In this study, we characterized a human neutralizing antibody 26D1 that is specific to HPV16. The monoclonal antibody 26D1 was isolated from a memory B cell of a volunteer donor who obtained three doses of an experimental HPV16/18 VLP vaccine^29^,^30^. Essential surface loops of the L1 proteins for 26D1 binding were identified by employing a series of HPV16/6 hybrid VLPs with surface loops swapped between two types to alter the epitope structure. Refined 26D1 epitope mapping was carried out by a second set of site-directed mutant HPV16 VLP proteins. The binding interface of 26D1 was predicted by homology modeling and molecular docking. Taken together, the results suggest that 26D1 recognizes a partially overlapping but distinct neutralizing epitope from that of H16.V5. This report is the first on the elicitation and epitope identification of a potent neutralizing antibody response against HPV16 VLPs in humans. Also the precise interpretation of neutralizing determinants provides a basis for prophylactic vaccine design.

## Results

### Isolation and characterization of a monoclonal antibody from an HPV16 L1 VLP vaccinee

A healthy female donor vaccinated with three doses of an experimental HPV16/18 L1 VLP vaccine in a Phase I clinical trial was selected for isolating HPV16-specific neutralizing antibodies (N-mAb) from individual memory B cells. As reported, antibodies cloned and expressed from singly sorted HPV 16-pseudovirus labeled memory B cells were predominantly IgG (>IgA>IgM), which could be assessed for expression, folding, and function (antigen binding and pseudovirus neutralization) *in vitro*^31^. Here, we screened 480 individual memory B cell culture supernatants from this donor for IgG1 positive (Supplementary Figure S1), binding to HPV16 VLPs and neutralizing HPV16 pseudovirus (PsVs). One IgG1 positive and antigen specific memory B cell clone was identified and further characterized, and the genes encoding an IgG were sequenced.

**Figure 1:**
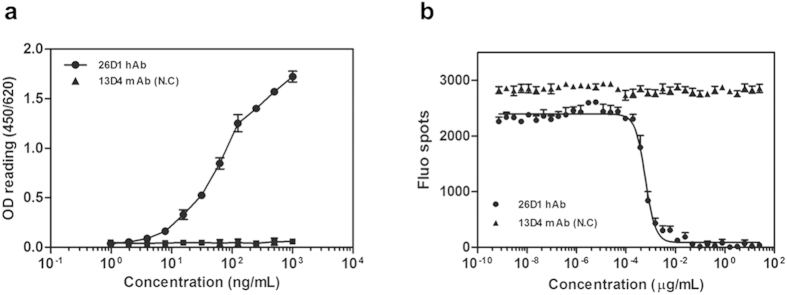
Specific binding to HPV 16 VLPs and neutralizing activity against pseudovirions of mAb 26D1. (**a**) Binding (EC_50_: 66 ng/ml) of 26D1 to HPV16 L1 VLPs in an antibody dose-dependent manner. (**b**) Neutralizing titers (NT_50_: 0.585 ng/ml) of 26D1 as measured by blocking HPV16 PsVs entry. The absorbance value (**a**) and fluorescent spot numbers (**b**) are indicated on the Y-axis, and antibody concentrations (**a**,**b**) are indicated on the X-axis. Each data point is the mean of three separate experiments. An influenza virus-specific 13D4 mAb was used as the negative control.

To characterize the human neutralizing antibody, the heavy- and light-chain immunoglobulin genes from this individual B cell well were amplified and cloned into IgG1 expression vectors, and the recombinant IgG1 antibody was named as 26D1. The V_H_ and V_L_ segments of 26D1 were assigned to IGHV4-39*01 and IGLV1-44*01 by IMGT, respectively. CDR sequences of 26D1 defined using the Kabat system^32^ are shown in Supplementary Table S1. Binding affinity and neutralization activity were calculated through four-parameter curve fittings (Fig. 1). The binding affinity of 26D1 to HPV16 VLPs shows an EC_50_ value of 66 ng/ml (EC_50_ binding is defined as the antibody concentration required to achieve 50% maximal binding, Fig. 1a). Neutralization activity of 26D1 to block PsVs entry has an NT_50_ value of 0.585 ng/ml (NT_50_ neutralizing activity is defined as the antibody concentration required to block viral entry by 50%, Fig. 1b). The K_D_ value of 26D1 binding to HPV16 VLPs as measured by Biacore was 6.2 × 10^−9^ M. Collectively, these results suggest that the human monoclonal antibody (mAb) 26D1 possesses a high affinity to HPV16 VLPs and potent viral neutralization activity.

### Inhibition of 26D1 and H16.V5 binding to HPV16 VLPs by heparan sulfate

**Figure 2:**
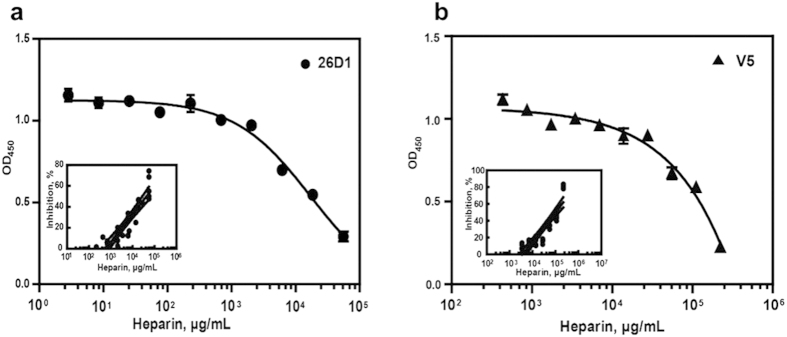
Inhibition of 26D1 (**a**) and H16.V5 (**b**) binding to HPV16 VLP by pre-binding of heparin. *Insets*: dose response curves showing the inhibition of 26D1 or H16.V5 binding by the soluble receptor molecule heparin in solution. The median inhibitory concentration (IC_50_) values for 26D1 (40 mg/ml) and H16.V5 (100 mg/ml) were approximated by linear regression fitting the data (inhibition rate vs. heparin concentration) from multiple runs (n = 6).

Specific binding of heparan sulfate, a cell surface receptor for HPV, to VLPs^33^ or PsV has been previously demonstrated^34^. The pre-binding of heparan sulfate to VLPs was shown to dramatically inhibit the binding of 26D1 and H16.V5 to VLPs in a concentration-dependent manner, as demonstrated by inhibition ELISA (Fig. 2). The IC_50_ values of 26D1 and H16.V5 for heparan sulfate in this inhibition assay are 40 mg/ml and 100 mg/ml, respectively (Fig. 2). In the inhibitory assay on HPV 16 and 31 PsVs, comparable IC_50_ value of ~4 mg/ml^35^ was observed for both serotypes of PsVs infecting HaCaT cells. The ~10 fold lower in IC_50_ value observed in this *in vitro* PsV infection model^35^ as compared to the VLP-based binding assay reported in this paper is likely due to much lower concentration of PsV particles was used when compared to the concentration of recombinant VLPs in the assays. The fact that soluble heparin sulfate can bind and inhibit the function of both PsVs and VLPs supports the notion that similar epitopes exist on PsVs and VLPs and heparin sulfate proteoglycan binding activity was maintained in both forms, analogous to authentic HPV virions.

### Identification of surface loops in HPV16 L1 VLP critical for 26D1 binding

**Figure 3:**
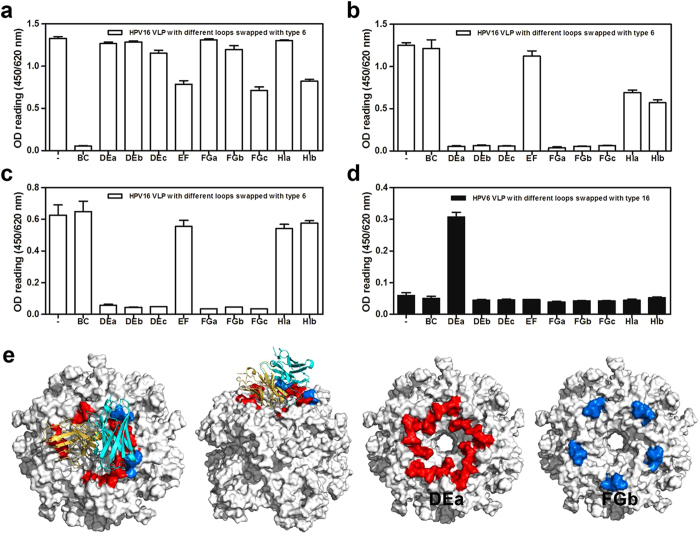
Identification of critical loops of the L1 protein for mAbs binding. Binding of a murine N-mAb (1A4) with BC loop specificity (**a**), H16.V5 (**b**) and 26D1 (**c**) to VLPs with HPV16 wild-type and HPV16 hybrid VLP mutants. VLP 16 wild-type (“-” on X-axis), HPV16 VLP with different loops swapped with the type 6 loops displayed as open bar. (**d**) Antibody 26D1 was tested by ELISA for reactivity to HPV6 wild-type and HPV6 hybrid VLP mutant. VLP 6 wild-type (“-” on X-axis), HPV6 VLP with different loops swapped with the type 16 loops displayed as solid bar. (**e**) Binding loop prediction by homology modeling and molecular docking. A model of 26D1 Fab is displayed as a solid ribbon diagram. The yellow and cyan ribbons represent the light chain and heavy chain, respectively. Predicted binding loops marked with red and blue are DEa loops and FGb loops. Residues involved in the 26D1 binding were mostly located at the DEa loop (red) and fewer at the FGb loop (blue).

To identify surface loops on VLPs that are critical for 26D1 binding, we established 20 HPV hybrid VLPs including 10 HPV16:6 hybrid VLPs that substituted HPV6 hypervariable loop residues (BC, DEa, DEb, DEc, EF, FGa, FGb, FGc, HIa, HIb loops) for HPV16 residues on the HPV16 L1 backbone, and another set of 10 different HPV6:16 hybrid VLPs that substituted HPV16 loop residues (BC, DEa, DEb, DEc, EF, FGa, FGb, FGc, HIa, HIb loops) for HPV6 sequences on the HPV6 L1 backbone (Supplementary Table S2, Supplementary Figure S2a). We tested the binding of 26D1, H16.V5 and another BC loop-binding mAb 1A4 to the 10 HPV16:6 hybrid VLPs (Fig. 3a–c). A murine N-mAb predominantly binding to BC loop (1A4) was used to confirm the structural integrity of hybrid VLPs in addition to overall particle morphology (Fig. 3a). The binding analysis showed that both H16.V5 (Fig. 3b) and 26D1 (Fig. 3c) reacted with HPV16 wild-type protein and the HPV16:6 constructs that contained BC, EF and HI loops replaced by type 6 homologous residues. The HPV16:6 constructs containing type 6-derived DE and FG loop substitutions lost binding activity to both 26D1 (Fig. 3c) and H16.V5 (Fig. 3b). With the exception of DE and FG loop, there was a significant decrease in H16.V5 binding to HPV16:6 hybrid VLP with HI loop substitution (Fig. 3b). It suggests that the DE and FG loops contain critical epitopes for both 26D1 and H16.V5 interaction to HPV16, and HI loop is also determined to be important antigenic region for H16.V5 interaction, albeit to a lesser extent, consistent with previous reports^12^. For the HPV6:16 hybrid VLPs binding, only the mutant expressing the type 16-derived DEa loop substitution was recognized by 26D1 (Fig. 3d), suggesting that compared to other regions of the DE loop and FG loops, the DEa loop of HPV16 L1 is the dominant antigenic region for 26D1 recognition.

The 26D1 interacting surface loops were further verified by molecular docking simulations^36^,^37^,^38^. The predicted immunocomplex structure was selected from poses with high scores in both ZDock and RDock calculations. In this model, due to steric hindrance, each pentamer on the VLP can only bind one 26D1 Fab at a given time. Contacting residues were mapped onto the DEa (residues 132–143) and FGb loops (residues 280–287) across five monomers and formed the top exit of the central tunnel on the pentamer (Fig. 3e). This result indicates that the DEa and FGb loops are in intimate proximity to each other and that the two loops constitute the conformational epitope for 26D1 binding, in agreement with the surface loop identification.

### Identification of key residues on the DEa loop for 26D1 binding

**Figure 4:**
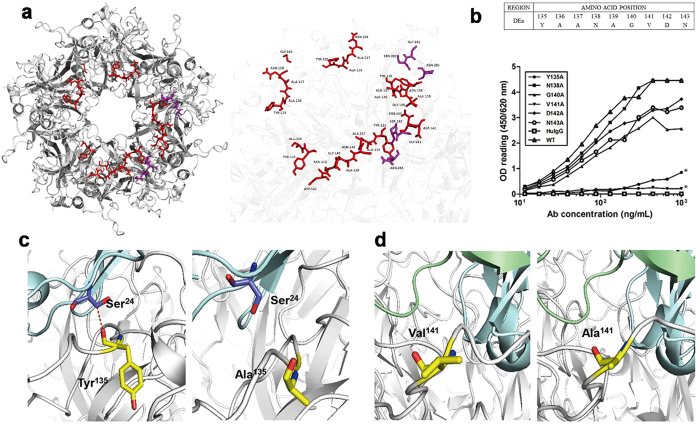
Identification of residues on the DEa loop that are critical for 26D1 binding. (**a**) Localization of the epitopes for 26D1 binding predicted by the docking models. Predicted binding residues were predominantly located on the DEa loop with a few on the FGb loop. The antigen-antibody complex is displayed as a solid ribbon diagram (*left*). Binding residues in the DEa loop and the FGb loop are rendered in ball-and-stick, colored in red and purple, respectively (*right*). (**b**) Binding of 26D1 to DEa loop mutants of HPV16 L1 VLPs. Alanine scanning mutagenesis was conducted on the DEa loop except the parental Ala residues. Binding of 26D1 to the six point-mutated VLPs was detected in a binding assay. HPV16 wild-type VLPs were used as a positive control, and a human IgG1 antibody was used as a negative control. Asterisks indicate a significant difference in binding. (**c**) Tyr^135^ forming hydrogen bond with Ser^24^ of 26D1 (*left*); the Y135A substitution resulted in hydrogen bond disappearance and a substantially lower affinity to 26D1 (*right*). (**d**) The side chain of Val^141^ on the DEa loop pointed to the interior of the proteins (*left*). The V141A substitution resulted in a hydrophilic interface (*right*), Y135A and V141A also led to an energy elevation of 0.64 kcal/mol and 0.79 kcal/mol, which is consistent with the reduced binding affinity. Binding residues are rendered in ball-and-stick, and residues of 26D1 Fab and DEa loop are marked with blue and yellow, respectively. Models of 26D1 Fab and HPV16 antigen are displayed as solid ribbon diagrams. The red dashed line between residues (Tyr^135^ on DEa loop, Ser^24^ on 26D1 Fab) represents a hydrogen bond.

The likely immunocomplex structure was further used to predict the key residues in conformation-dependent antibody binding. As shown in Fig. 4a, 8 amino acids at positions 135–140 (YAANAG) and 142–143 (DN) of the DEa loop, 2 amino acids (GS) at positions 281–282 of the FGb loop, and residue Asn (N) at position 285 of the FGb loop were predicted to be conformational epitopes across two adjacent L1 monomers (Fig. 4a, Supplementary Table S3). A linear epitope in the vicinity was found on the DEa loop, which takes up position 135–136 (YA). Furthermore, the other two monomers in the counter-clockwise direction also contained a consecutive four amino acid epitope (DEa loop position 135–138, YAAN), which was recognized by 26D1. In addition, Gly^140^ on one of these two DEa loops was also predicted to be involved in 26D1 binding (Fig. 4a, Supplementary Table S3). By examining the formation of predicted hydrogen bonds, most of the key residues were located on the DEa loop, with a few on the FGb loop (Supplementary Table S3), indicating that the residues on the DEa loop have a significant influence on the binding of 26D1 to the HPV16 L1 VLPs.

The likely structure also suggests that residues 135 to 143 on the DEa loop are mostly involved in specific interaction with 26D1. We generated and purified six point-mutated HPV16 VLPs for these residues using site-directed mutagenesis (Supplementary Figure S2b). The individual residues of the DEa loop (residues 135–143) (Y^135^, N^138^, G^140^, V^141^, D^142^, N^143^), all except three parental Ala residues, were successively mutated to Ala, which did not impair the formation of VLPs (Fig. 4b). The results showed that the VLP with the N138A mutation maintained 26D1 binding ability. The VLPs with G140A, D142A and N143A mutations demonstrated reduced 26D1 interaction, which led to slightly reduced binding affinity. In contrast, the VLPs with Y135A and V141A mutations exhibited drastically decreased binding to 26D1 to 3% and 5% of the wild-type VLP binding level, respectively (Fig. 4b).

The structures of VLPs with different mutations were also modelled by *in silico* mutagenesis. Based on the atomic model of the immune complex, the key residue Tyr^135^ forms a hydrogen bond with Ser^24^ from 26D1 (Fig. 4c, Supplementary Table S3). Although the hydrogen acceptor sits at the main chain, loss of the phenolic group in the Y135A mutation results in a collapse of the surface of the epitope. This local structure alteration may contribute to the reduced affinity of 26D1 to the epitope, and this prediction was confirmed by the binding assay on mutated VLPs (Fig. 4b,c). Val^141^ on the DEa loop might contribute minimally to the predicted antibody-antigen interaction, but the VLP with the V141A mutations showed a significant loss of 26D1 binding (Fig. 4b). As indicated in Fig. 4d, the hydrophobic residue valine, with its side chain pointing to the interior of the proteins, might play a role in maintaining the local surface conformation. Thus, the V141A mutation may also induce a surface collapse, resulting in reduced affinity. Based on the structure of *in silico* mutagenesis, the free energy shift could be calculated to evaluate the change in binding affinity before and after the mutation. The Y135A and V141A mutations led to an energy increase of 0.64 kcal/mol and 0.79 kcal/mol, respectively, which stands for the potential deprivation of binding affinity (Fig. 4d).

### Epitope mapping using surface plasmon resonance

**Figure 5:**
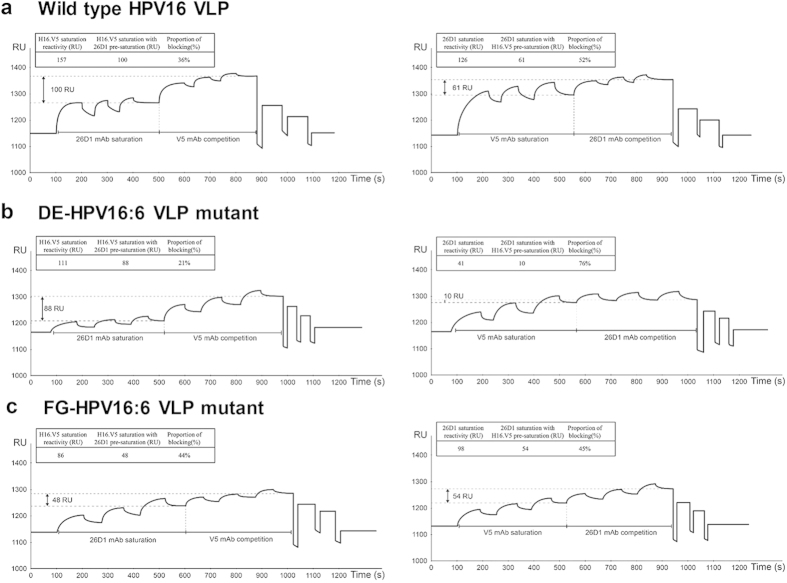
Surface plasmon resonance of a pairwise binding study for cross inhibition between 26D1 and H16.V5. (**a**) Epitope competition between 26D1 and H16.V5 on binding to wild type HPV16 VLPs which were covalently attached to a sensor chip. The saturation units for 26D1 and H16.V5 (100 μg/ml, injected three times) were 126 RU and 157 RU, respectively. Epitope competition was established by pre-saturation of the surface by 26D1 followed by injecting a saturating amount of H16.V5. With pre-saturation of 26D1, the H16.V5 saturation level was 100 RU, corresponding to a 36% reduction of the original H16.V5 saturation binding (left). We reciprocally assessed 26D1 saturated binding level with H16.V5 pre-saturation, which resulted in 52% reduction of the original 26D1 saturated level (right). (**b**) Epitope competition between 26D1 and H16.V5 on binding to DE-HPV16:6 VLP mutant (HPV16 VLPs with DE loops swapped with the type 6 loops). The saturation units for 26D1 and H16.V5 were 41 RU and 111 RU, respectively. 21% of H16.V5 binding epitopes were inhibited with 26D1 pre-saturation (left), and 76% of 26D1 binding epitopes were blocked by H16.V5 pre-saturation (right). (**c**) Epitope competition between 26D1 and H16.V5 on binding to FG-HPV16:6 VLP mutant (HPV16 VLPs with FG loops swapped with the type 6 loops). The saturation units for 26D1 and H16.V5 were 98 RU and 86 RU, respectively. After 26D1 pre-saturation, the saturated binding level for H16.V5 was 48 RU and resulted in 44% reduction of the H16.V5 saturation binding (left). Reciprocally, 45% of 26D1 binding epitopes were blocked by H16.V5 pre-saturation (right). The saturation resonance units (RU) for mAbs with or without pre-saturation and blocking proportion are indicated in the figure.

Epitope competition between 26D1 and H16.V5 on binding to HPV16 VLPs was performed using Biacore surface plasmon resonance-based technology. The saturation binding levels of 26D1 and H16.V5 to the immobilized wild type VLPs on the chip were measured to be 126 RU and 157 RU, respectively. Then, antibody binding competition was assessed by pre-saturating the surface with 26D1 and successively injecting a saturating amount of H16.V5. The saturated binding level for H16.V5 with pre-saturation of 26D1 was 100 RU, which is a 36% reduction of the original H16.V5 saturation binding (157 RU) (Fig. 5a, left). To evaluate the inhibition rate of 26D1 by H16.V5, we reciprocally assessed 26D1 saturated binding level with H16.V5 pre-saturation, which resulted in 52% reduction of the original 26D1 saturated level (126 RU) (Fig. 5a, right). This result suggests that the epitopes recognized by 26D1 and H16.V5 partially overlap, and higher proportion of 26D1 epitopes can be blocked by pre-saturation of H16.V5.

HPV16 VLPs with DE (DE-HPV16:6 VLP mutant) and FG loops (FG-HPV16:6 VLP mutant) swapped with the type 6 loops were also used to analyze competition reactivity between 26D1 and H16.V5. For DE-HPV16:6 VLP mutant binding on the chip, the saturation reactivity of 26D1 was dramatically reduced to be 41 RU, and 76% of these 26D1 binding epitopes were blocked by H16.V5 pre-saturation (111 RU) (Fig. 5b, right). In contrast, only 21% of H16.V5 binding epitopes were inhibited with 26D1 pre-saturation (41 RU) (Fig. 5b, left). This result obtained indicates that 26D1 binding reactivity is greatly affected by DE loop substitution which is in agreement with the epitope identification and structural analysis. When H16.V5 binding to FG-HPV16:6 VLP mutant on the sensor chip, the saturated binding level for H16.V5 was 48 RU after 26D1 pre-saturation, resulted in 44% blocking percentage by 26D1 (Fig. 5c, left). Reciprocally, 45% of 26D1 binding epitopes were blocked by H16.V5 pre-saturation directed against FG-HPV16:6 VLP mutant (Fig. 5c, right). The data suggests that the difference in 26D1 and H16.V5 binding is primarily in the specific interaction with DE loop.

The capacity of 26D1 to block the reactivity of human sera to HPV16 VLPs was evaluated using a panel of 16 human serum samples. Eight patient samples and eight serum samples from vaccinees, who were previously determined to be seropositive in a binding assay to HPV16 VLPs, were selected at random. The biosensor method was also used for this inhibition assay. Among the eight naturally HPV-infected serum samples, 26D1 was shown to completely block the reactivity of three samples to HPV16 capsids (Table 1). In contrast, no vaccinated serum samples were completely blocked by 26D1 (Table 1). For the other five patient samples, 26D1 demonstrated a partial blockage of HPV16 capsid binding ranging from 65% to 93%. Strong (>80%) and intermediate blocking activity (≥60–80%) accounted for 25% (n = 2) and 38% (n = 3) of the 8 patient serum samples, respectively (Table 1). The average inhibition rate for the 8 patient samples was 86%, and the blocking activity of 26D1 to the 8 vaccinated serum samples was observational lower (mean = 70%, Table 1). Only two vaccinee serum samples were strongly inhibited by 26D1 (>80%), four samples were found to have intermediate inhibition activities (≥60–80%), and 26D1 showed low blocking activity for the remaining two samples from the vaccinees (<60%, Table 1). The capacity of 26D1 was observed to block both the reactivity of serum samples from naturally infected individuals and vaccinees, suggesting that the epitope recognized by 26D1 is immunodominant.

### Overlapping epitopes between 26D1 and H16.V5

**Figure 6:**
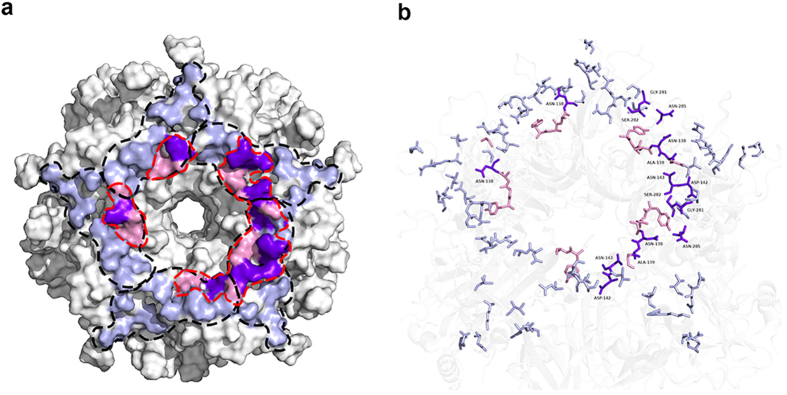
Identification of partially overlapping but distinct neutralizing epitopes of H16.V5 by 26D1. (**a**) The footprint of H16.V5 and 26D1 Fab in the atomic model. H16.V5 Fab binds five loops across two L1 proteins that are adjacent to one another^12^. 26D1 Fab binds across five monomers adjacent to the central tunnel. The boundary of each V5 Fab-binding residue is marked with black dashed lines^12^. Each pentamer on VLP can only bind one 26D1 Fab at a time due to steric hindrance. The boundary of the 26D1 Fab-binding residues is marked with red dashed lines. As reported, complete H16.V5 epitopes were identified that map to 17 residues of multiple loops (BC, FG, DE and HI loops) from two neighboring L1 proteins^12^. Based on this result, the contacting residues of V5 mAb and 26D1 human mAb on the capsomeric surface are highlighted in blue and pink, respectively. Overlapping residues (N^138^, A^139^, D^142^, N^143^, G^281^, S^282^, N^285^) of H16.V5 and 26D1 are highlighted in purple. (**b**) Contacting residues of H16.V5 and 26D1 are rendered in ball-and-stick and are colored blue and pink, respectively. Overlapping residues are labeled and colored purple.

Mapping the epitopes onto the roadmap of a single capsomere revealed the similarities and differences between H16.V5 and 26D1 (Fig. 6). Of the 17 binding residues targeted by H16.V5, 13 residues are on the BC, FG and DE loops of the first L1 protein (Gln^181^, Asp^184^ on the BC loop; Val^141^, Asp^142^ and Asn^143^ on the DE loop; Val^267^, Gln^269^, Asn^270^, Ser^280^, Gly^281^, Ser^282^, Thr^283^ and Asn^285^ on the FG loop), and four residues are on the HI and DE loops on the second neighboring L1 protein (Thr^358^, Lys^361^ on the HI loop; Asn^138^, Ala^139^ on the DE loop)^12^. Five loops presented by the two L1 proteins consecutively across the footprint form a left-right-left-left-right L1 configuration, conferring a stabilizing force to the capsid (Fig. 6a)^12^. A similar study also demonstrated that seven residues (Asn^138^.DE, Ala^139^.DE, Gln^181^.EF, Ser^282^.FG, Asn^285^.FG, Ile^348^.HI, and Lys^361^.HI) were essential components in the common epitopes interacting with four other HPV16 N-mAbs^39^. In contrast, the epitope of 26D1 in the model encircles the exposed surface around the rim of the central tunnel exit along the DE loop track. Some regions of the DE loop are juxtaposed with the binding region of the FG loop (Figs 3e and 6a). Superimposing the two complex structures predicted that four binding residues of the DEa loop (Asn^138^, Ala^139^, Asp^142^, Asn^143^) and three residues of the FGb loop (Gly^281^, Ser^282^, Asn^285^) were overlapping epitopes targeted by both H16.V5 and 26D1, four of which (Asn^138^.DE, Ala^139^.DE, Ser^282^.FG, Asn^285^.FG) were included in the common epitopes^39^ mentioned above (Figs 4a and 6b, Supplementary Table S3). Moreover, two residues (Asn^138^ and Ser^282^) on this patch were predicted to form a hydrogen bond in the paratope of 26D1 (Fig. 6b).

## Discussion

The primary objective of a prophylactic HPV16 L1 VLP-based vaccine is to develop protective immunity against viral infection, which in large part depends on elicitation of neutralizing antibodies^40^. Memory B cells help to sustain antibody levels over time by rapidly differentiating into antibody secreting cells upon pathogen re-exposure. Immunological memory that forms following HPV VLP vaccination remains elusive, until recently Scherer and colleagues characterized the memory B cells elicited by HPV vaccine. Their findings suggest that HPV vaccine provides an excellent model for studying the development of B cell memory^31^. In this study, we established a robust memory B-cell culturing protocol to drive proliferation and differentiation to antibody producing cells *in vitro*, which allowed for functional screening of potent neutralizing antibody from an HPV VLP vaccinated individual. In addition, due to the limited information on neutralizing epitopes on the HPV16 capsid protein, we isolated a specific neutralizing antibody to characterize their immunoreactive epitopes to further improve our understanding of L1 topography as well as providing unique tools for tracking important antibody specificities generated by natural infection and vaccination. This is also the first report on the identification of a human monoclonal antibody recognizing a distinct neutralizing epitopes on the HPV16 capsid protein.

The neutralization epitopes of HPV16 are usually detected with murine monoclonal antibodies such as H16.V5. An epitope composed of the FG and HI loops identified by H16.V5 has been reported to be immunodominant for HPV16^23^,^27^,^41^,^42^. However, a series of experiments have suggested that there may be additional critical neutralizing epitopes on the capsid protein during human natural HPV16 infection^43^,^44^,^45^. The complete H16.V5 epitope has recently been mapped through a cryo-electron microscopy study to a total of 17 residues of multiple loops (BC, FG, DE and HI loops) from two neighboring L1 proteins. Among the 17 residues, eight of them were found on the FG loop, five were found on the DE loop, and two were located on the HI loop^12^, which is in agreement with our surface loop identification that DE and FG loops were major component for H16.V5 recognition, and HI loop had minor impact on H16.V5 binding. These findings validate our conclusion based on the human neutralizing antibody 26D1 studies that in addition to the FG loop, the DE loop on HPV16 on the capsid protein is also one of the most important regions for neutralizing activity. As measured by epitope competition, although the epitopes of H16.V5 and 26D1 are overlapping, the two epitopes are distinct and their binding difference is mainly located on DE loop. Epitope identification also confirmed that 26D1 recognized epitope is more dependent on residues on the DE loop rather than the ones on the FG loop where the H16.V5 contact residues reside^12^,^39^.

The structural determination and homology modeling of the VLP-Fab complex allowed the predicted interpretation of the similarity and difference between H16.V5 and 26D1 epitopes^12^,^39^,^42^. But it should be noted that these observations were based on structure analysis performed by crystallographic software, the authentic residue interaction will be further investigated through 26D1 Fab co-crystallization with VLP or L1 pentamer. The critical role of residue Tyr^135^ makes the 26D1-defined epitope distinctively different from that of H16.V5, as this residue was not shown to be involved in the H16.V5 epitope. Although this residue has been shown to recognize another two N-mAbs, namely H16.1A and H16.263A2^39^. This distinct 26D1-defined epitope structure explains not only the elicitation of a strong immune response in humans against this antigenic site, but it also provides a molecular proposition for VLPs or PsVs to recognize human HPV16-specific neutralizing antibodies.

Both H16.V5 and 26D1 recognize immunodominant epitopes. Based on the antibody footprinting results, 26D1 epitope is an immunodominant epitope as demonstrated with over 70% competition of polyclonal antibodies in patient sera or vaccine sera by 26D1. More sera samples and WHO standard sera to HPV16^46^ will be involved in competition assay of 26D1 to better define the competition levels in serum samples. In addition, H16.V5 was proven to protect from PsV challenge from murine genital model^47^, whether 26D1 affords protection from genital infection *in vivo* needs to be further studied using this model. The 26D1 epitope was impaired by prior binding of heparan sulfates or heparin, which is a soluble receptor molecule present on the cell surface as highly negatively charged chains in proteoglycans^33^,^34^,^48^. Both heparan sulfate and H16.V5 were shown to bind the capsomere from the top of the pentamer^42^. The impairment of N-mAb binding by heparan sulfate was due to either steric hindrance or conformational changes induced by heparin binding, a process that is important for viral cell entry. This observation is consistent with the fact that heparin in solution or neutralizing antibodies such as 26D1 and H16.V5 can efficiently neutralize the HPV pseudovirus, mostly likely by blocking the cell entry process.

In summary, a human HPV16 neutralizing antibody 26D1 was isolated from a vaccinee through a B-cell cloning procedure. Its epitope was mapped in details through loop swapping and site-directed mutagenesis of the major antigenic sites. The 26D1 epitope consists of residues from the FG and DE loops, predominantly in the DEa loop, with the Tyr^135^ and Val^141^ in the DEa loop being the most critical residues. Pairwise epitope mapping and analysis of key residues at the Ag-Ab interface revealed that 26D1 recognizes a conformational epitope that partially overlaps that of H16.V5. These findings contribute to the improved our understanding of the antigenic structure of the HPV16 L1 VLPs and of human B-cell epitopes in the immune responses to HPV natural infection or vaccination. The work based on a human IgG elicited by vaccination provided better understanding of type-specific neutralizing epitopes for HPV 16. Such an understanding on structural basis of neutralizing epitopes will aid future vaccine design to induce cross-type protection with hybrid VLPs by specific loop grafting across different types, thus to afford broad spectrum protective immunity.

## Materials and Methods

### Ethics statement

The vaccination and blood sampling of the healthy volunteer strictly adhered to the guideline and were compliant with the clinical protocol, which was approved by the Xiamen University Institutional Review Board. Written informed consent was obtained from the donor for use of the serum sample. Independent Ethics Committee approval was obtained from the Ethics Committee of the National Institute of Diagnostics and Vaccine Development in infectious diseases.

### Study subject and samples

A healthy female volunteer was recruited from a Phase I clinical trial of an experimental HPV16/18 vaccine^29^,^30^. The serum HPV16 neutralization titer (NT) for the subject at the point of sampling was 40,960 (dilution factor), representing a strong immune response after three doses of HPV16/18 L1 VLP vaccination.

Eight serum samples of vaccines were collected from a Phase I clinical trial of the HPV16/18 vaccine, and four patient serum samples were collected from donors diagnosed with cervical intraepithelial neoplasia (CINII); three patients were diagnosed with low-grade squamous intraepithelial lesion (LSIL), and one patient sample was diagnosed with high-grade squamous intraepithelial lesion (HSIL). All serum samples were determined to be seropositive for antibody binding activity against HPV16 VLPs.

### Memory B-cell culture, isolation and antibody cloning

A total of 40 ml of whole blood from the female vaccinee was collected for peripheral blood mononuclear cell (PBMC) isolation. IgG^+^ memory B cells were isolated from the PBMCs using the EasySep™ Human Memory B Cell Isolation Kit (Stemcell, Vancouver, CA). To promote memory B-cell differentiation into antibody-secreting plasma cells, memory B cells were seeded at an average of 1.5 B cells/well in 96-well plates in the presence of 50 Gy irradiated CD40 ligand feeder cells and 50 ng/ml recombinant human IL-21 protein (Sino Biologicals, Beijing, China). The culture supernatants were collected 14 days later and screened for binding activity to the HPV16 VLPs and neutralizing activity in the HPV16 pseudovirus (PsVs)-based neutralization. Total RNA was isolated from positive lysed B-cell cultures using an RNeasy Micro kit (Qiagen, Valencia, CA), and reverse transcription was carried out using the SuperScript III First-Strand Synthesis SuperMix kit and specific IgG1 reverse primer (Invitrogen, Carlsbad, CA). Heavy and light chain variable regions were then amplified by PCR using IgG1 V_H_, V_K_, V_λ_ family-leader region-specific primers^49^ and sub-cloned into the human IgG1 expression vectors pTT5-HC with a heavy chain constant region and pTT5-LC with a light chain constant region, respectively. The recombinant vectors were co-transfected into CHO cells at a 1:1 ratio using polyethylenimine (PEI; Polyscience, Chicago, IL) and cultured for 7 days. Antibody IgG1 was purified from culture supernatants by protein A affinity chromatography (General Electric Company, Pittsburgh, PA).

### IgG1 detection of memory B cell cultures

The concentration of IgG1 in B cell culture supernatants was assessed from 2 plates randomly chosen from total of 5 plates in ELISA assay. Maxisorp Nunc-Immuno Plates (Thermo Scientific, Waltham, MA) were coated with 50 μl per well of goat anti-human IgG1 (Fc) antibody (Thermo Scientific, Waltham, MA) at 4 μg/ml in PBS at 4 °C overnight. 50 μl per well of B cell culture supernatants was then added. Afterwards, 50 μl per well of HRP-conjugated goat anti-Human IgG1 (Southern Biotech Inc, Birmingham, AL) at 1:2,000 dilution was added as secondary antibody. Reaction was read at 450 nm on VICTOR Multilabel Counter (Wallac/Perkin Elmer, Waltham, MA). Purified human IgG1 whole molecule of known concentration (Pierce, Rockford, IL) with 3 fold dilutions starting at 10 μg/ml was used as standard curve.

### ELISA binding and pseudovirus neutralization assays

Intact HPV16 capsids and hybrid VLPs (3 μg/ml) were coated onto 96-well microtiter plates (Corning, Glendale, AZ) in 20 mM phosphate at pH 7.4 and 0.3 M NaCl, and 100 μl B cell culture supernatants were added. For EC_50_ and hybrids binding measurements, purified antibody was initially diluted at 1 μg/ml followed by serial dilution (1:2) across a polypropylene 96-well plate in PBS. The EC_50_ titers represent the concentration for 50% of maximal binding. We chose the OD reading of 26D1/H16.V5/1A4 at concentration of 12 ng/ml for surface loop identification in hybrid VLPs binding assay.

HPV16 pseudo-viruses were produced according to previous studies^50^,^51^,^52^. HPV16 pseudovirions vector coupled to a green fluorescent protein reporter gene (pN31-GFP) were kindly provided by Dr. J. T. Schiller^53^. Neutralization procedure has been described in our previous report^54^. Firstly, 293FT cells were harvested 72 h after transfection, lysed with cell lysis buffer and incubated at 37 °C for 24 h. Afterwards, 5 M NaCl solution was added to the samples to extract the cell lysates. TCID50 (tissue culture infective dose) of the supernatant was then measured to determine the titers of the PsVs^55^. 293FT cells were incubated at 37 °C in 96-well plate at a density of 1.5 × 10^4^ cells per well for 6 h. 26D1 were subjected to a 2-fold dilution. PsVs were diluted to 2 × 10^5^ TCID50/μl. Sixty μl of the PsV diluent and 60 μl of the serially diluted antibody were mixed and incubated at 4 °C for 1 h. The negative control was prepared by mixing 60 μl of the PsV diluent with 60 μl of the culture medium. Then, 100 μl of the above mixtures were added designated wells and incubated at 37 °C for 72 h. Flow cytometry was used to detect the number of HPV-pseudovirus-infected enhanced green fluorescent protein (EGFP)-expressing cells. The endpoint titers were calculated as the log_10_ of the highest dilution with a percent infection inhibition higher than 50%.

### Determination of 26D1 binding affinity by surface plasmon resonance

Affinity measurement was conducted at 25 °C on a Biacore 3000 instrument (GE Healthcare, Uppsala, Sweden). Mouse anti-human IgG (25 μg/ml) was directly immobilized to the surface of a CM5 sensor chip via covalent amine coupling. HPV16 VLP protein was initially diluted as 20 nM and then serial diluted to five concentrations (0.625, 1.25, 2.5, 5, 10, 20 nM) in 250 μl by using 0.5 M NaCl, 5 mM histidine buffer, 0.02% PS80, pH 6.2. Next, 5 μg/ml of 26D1 was loaded onto sensor chip for 60 s at a flow rate of 5 μl/min. Then each concentration of HPV16 VLP analyte was loaded onto sensor chip to carry out an association step with 26D1 ligand for 120 s for determining their relative on-rates; this was followed by a dissociation step for 600 s for determining their relative off-rates. 10 mM glycine-HCl (pH 1.7) was used as regeneration buffer at a flow rate of 10 μl/min for 30 s. The sensorgram was recorded and subjected to reference and buffer subtraction and then evaluated using BIAevaluation software.

### VLP binding competition between 26D1 and H16.V5 by surface plasmon resonance

The analysis was performed with a Biacore 3000 (GE Healthcare, Uppsala, Sweden) equipped with CM5 sensor chips. The HPV16 L1 VLPs (20 μg/ml in 400 μl of PBS buffer) were covalently coupled to the CM5 sensor surface at a flow rate of 5 μl/min. Antibody saturation of the bound HPV16 L1 VLPs was achieved for 26D1 after three injections of 100 μg/ml antibody diluted in 50 μl PBS. VLP binding competition between 26D1 and H16.V5 was established by successively injecting H16.V5 three times at 100 μg/ml diluted in 50 μl PBS to the 26D1 saturated sensor surface. Similarly, we reciprocally assessed saturated binding level for 26D1 with H16.V5 pre-saturation. Then, HPV16:6 hybrid VLPs that substituted HPV6 hypervariable DE and FG loop were respectively loaded on the sensorchip for epitope competition between 26D1 and H16.V5, as described above.

Eight patient sera and eight serum samples from vaccinees with different neutralization titers were randomly selected for 26D1 inhibition study. Sera blocking experiments were conducted with the saturation amount of serum after 26D1 blocking as described above. Binding was measured as a change in resonance units (RU) on the chip. Bovine serum albumin was used as a control for nonspecific binding.

### Homology modeling and molecular docking

The initial 26D1 3D structure model was generated using the Homology Module of Discovery Studio 4.1 (Accelrys Inc.) using the CDR sequence information listed in Supplementary Table S2. The HPV16 L1 pentamer (PDB: 2R5H)^8^ and 26D1 structure were submitted to the ZDock module for molecular docking analysis. Two previously described consecutive steps of calculations, that is, geometry search and energy search^56^, were run on the ZDock and RDock programs^36^,^38^. The top 50 poses from the ZDock results were selected for RDock evaluation. The pose with the highest score in both the ZDock and RDock calculations was selected as the predicted complex^57^. The 26D1 epitope was then concluded from the contact region between the pentamer and the antibody. All structures were optimized in Discovery Studio, and the energy of interaction was calculated using CHARMM force field.

### Generation of hybrid VLPs and VLPs with mutated amino acids

Type-specific amino acids in hypervariable loops of the HPV16 and HPV6 L1 capsid proteins were identified by CLUSTAL amino acid sequence alignment (Supplementary Table S2)^58^. To generate HPV16:6 hybrid virus-like particles, ten hypervariable loop sequences (BC, DEa, DEb, DEc, EF, FGa, FGb, FGc, HIa, HIb; Supplementary Table S2) of the HPV16 L1 capsid protein were exchanged by the corresponding amino acid residues of the HPV6 L1 protein on the HPV16 L1 backbone. HPV6:16 hybrids that substituted HPV16 loop sequences for HPV6 residues on the HPV6 L1 backbone were generated reciprocally. The construction of hybrid VLP mutants were performed as previously described^54^.

### Competitive inhibition of 26D1 and H16.V5 by heparin

The human monoclonal antibodies 26D1 and H16.V5 were used for the heparin competitive binding ELISA. Briefly, HPV16 VLP was coated on a 96-well plate overnight at 4 °C. After HPV16 VLP coating, a total of 100 μl of 55.55 mg/ml heparin was added to the first well and a 2-fold serial dilution was used for the following wells in the column. After a 30-min incubation at room temperature, 200 ng/ml 26D1 was added to each well and incubated for 1 h. The plate was washed 5 times, and goat anti-human IgG-HRP diluted in enzyme dilution buffer was then added, followed by incubation for 1 h. After washing 5 times, the plate was incubated with a solution of tetramethylbenzidine substrate for 10 min. The reaction was stopped with 2 M H_2_SO_4_. The optical density was measured spectrophotometrically at 450 nm.

### Transmission electron microscopy (TEM)

Approximately 15 μl of HPV16 L1 VLPs or hybrid VLPs at 200 μg/ml was absorbed onto carbon-coated copper grids, blotted dry, and stained with freshly filtered 2% phosphotungstic acid (pH 6.4). Grids were examined under the FEI Tecnai T12 TEM at an accelerating voltage of 120 kV and photographed at a nominal 25,000 × magnification.

## Additional Information

**How to cite this article**: Xia, L. *et al*. A human monoclonal antibody against HPV16 recognizes an immunodominant and neutralizing epitope partially overlapping with that of H16.V5. *Sci. Rep*. **6**, 19042; doi: 10.1038/srep19042 (2016).

## Supplementary Material

Supplementary Information

## Figures and Tables

**Table 1:** HPV16 capsid immunoreactivity of human sera from naturally infected individuals or vaccinees and the percentage of 26D1 inhibition in a surface plasmon resonance-based binding assay against HPV16 VLPs.

Serum No.	Diagnosis	Neutralization titer (dilution factor)	Blocking percentage (%)
73	CINII	200	100
178	LSIL	200	100
121	LSIL	200	100
196	HSIL	2,000	93
75	CINII	200	84
158	CINII	200	75
72	LSIL	200	70
139	CINII	200	65
8	VLP vaccination	40,960	95
3	VLP vaccination	20,480	81
17	VLP vaccination	20,480	79
6	VLP vaccination	20,480	70
23	VLP vaccination	20,480	66
28	VLP vaccination	40,960	60
15	VLP vaccination	20,480	56
5	VLP vaccination	40,960	56
Inhibition percentage (mean) of HPV16		Patient sera	86% ± 21%
seropositive sera by 26D1		VLP vaccination sera	70% ± 25%

CINII, cervical intraepithelial neoplasia; LSIL, low-grade squamous intraepithelial lesion; HSIL, high-grade squamous intraepithelial lesion.

## References

[b1] Stern P. L. . Therapy of human papillomavirus-related disease. Vaccine. 30 Suppl 5, F71–82, doi: 10.1016/j.vaccine.2012.05.091 (2012). 23199967 PMC4155500

[b2] Forman D. . Global Burden of Human Papillomavirus and Related Diseases. Vaccine. 30, Supplement 5, F12–F23, doi: 10.1016/j.vaccine.2012.07.055 (2012). 23199955

[b3] Parkin D. M. & Bray F. Chapter 2: The burden of HPV-related cancers. Vaccine. 24, Supplement 3, S11–S25, doi: 10.1016/j.vaccine.2006.05.111 (2006). 16949997

[b4] Stanley M. Immunobiology of HPV and HPV vaccines. Gynecol. oncol. 109, S15–21, doi: 10.1016/j.ygyno.2008.02.003 (2008). 18474288

[b5] Hagensee M. E., Yaegashi N. & Galloway D. A. Self-assembly of human papillomavirus type 1 capsids by expression of the L1 protein alone or by coexpression of the L1 and L2 capsid proteins. J. Virol. 67, 315–322 (1993). 8380079 10.1128/jvi.67.1.315-322.1993 PMC237365

[b6] Kirnbauer R., Booy F., Cheng N., Lowy D. R. & Schiller J. T. Papillomavirus L1 major capsid protein self-assembles into virus-like particles that are highly immunogenic. Proc. Natl. Acad. Sci. USA 89, 12180–12184 (1992). 1334560 10.1073/pnas.89.24.12180 PMC50722

[b7] Kirnbauer R. . Efficient self-assembly of human papillomavirus type 16 L1 and L1-L2 into virus-like particles. J. Virol. 67, 6929–6936 (1993). 8230414 10.1128/jvi.67.12.6929-6936.1993 PMC238150

[b8] Bishop B. . Crystal structures of four types of human papillomavirus L1 capsid proteins: understanding the specificity of neutralizing monoclonal antibodies. J. Biol. Chem. 282, 31803–31811, doi: 10.1074/jbc.M706380200 (2007). 17804402

[b9] Schiller J. T. & Lowy D. R. Papillomavirus-Like Particle Vaccines. JNCI Monographs. 2000, 50–54 (2000). 10.1093/oxfordjournals.jncimonographs.a024258 11158207

[b10] Viscidi R. P. . Prevalence of antibodies to human papillomavirus (HPV) type 16 virus-like particles in relation to cervical HPV infection among college women. Clin. Diagn. Lab. Immun. 4, 122–126 (1997). 10.1128/cdli.4.2.122-126.1997 PMC170489 9067643

[b11] Buck C. B., Pastrana D. V., Lowy D. R. & Schiller J. T. Efficient Intracellular Assembly of Papillomaviral Vectors. J. Virol. 78, 751–757, doi: 10.1128/jvi.78.2.751-757.2004 (2004). 14694107 PMC368835

[b12] Lee H. . A cryo-electron microscopy study identifies the complete H16.V5 epitope and reveals global conformational changes initiated by binding of the neutralizing antibody fragment. J. Virol. 89, 1428–1438, doi: 10.1128/JVI.02898-14 (2015). 25392224 PMC4300654

[b13] Breitburd F. . Immunization with viruslike particles from cottontail rabbit papillomavirus (CRPV) can protect against experimental CRPV infection. J. Virol. 69, 3959–3963 (1995). 7745754 10.1128/jvi.69.6.3959-3963.1995 PMC189126

[b14] Jansen K. U. . Vaccination with yeast-expressed cottontail rabbit papillomavirus (CRPV) virus-like particles protects rabbits from CRPV-induced papilloma formation. Vaccine. 13, 1509–1514, doi: 10.1016/0264-410X(95)00103-8 (1995). 8578834

[b15] Christensen N. D., Reed C. A., Cladel N. M., Han R. & Kreider J. W. Immunization with viruslike particles induces long-term protection of rabbits against challenge with cottontail rabbit papillomavirus. J. Virol. 70, 960–965 (1996). 8551636 10.1128/jvi.70.2.960-965.1996 PMC189900

[b16] Mukherjee S., Thorsteinsson M. V., Johnston L. B., DePhillips P. A. & Zlotnick A. A Quantitative Description of *In Vitro* Assembly of Human Papillomavirus 16 Virus-Like Particles. J. Mol. Biol. 381, 229–237, doi: 10.1016/j.jmb.2008.05.079 (2008). 18585738

[b17] Suzich J. A. . Systemic immunization with papillomavirus L1 protein completely prevents the development of viral mucosal papillomas. Proc. Natl. Acad. Sci. USA 92, 11553–11557 (1995). 8524802 10.1073/pnas.92.25.11553 PMC40440

[b18] Chen X. S., Garcea R. L., Goldberg I., Casini G. & Harrison S. C. Structure of Small Virus-like Particles Assembled from the L1 Protein of Human Papillomavirus 16. Mol. Cell. 5, 557–567, doi: 10.1016/S1097-2765(00)80449-9 (2000). 10882140

[b19] Yaegashi N. . Characterization of murine polyclonal antisera and monoclonal antibodies generated against intact and denatured human papillomavirus type 1 virions. J. Virol. 65, 1578–1583 (1991). 1847474 10.1128/jvi.65.3.1578-1583.1991 PMC239941

[b20] Heino P. . Human papillomavirus type 16 capsids expose multiple type-restricted and type-common antigenic epitopes. J. Gen. Virol. 76, 1141–1153, doi: 10.1099/0022-1317-76-5-1141 (1995). 7537325

[b21] Rose R. C., Bonnez W., Da Rin C., McCance D. J. & Reichman R. C. Serological differentiation of human papillomavirus types 11, 16 and 18 using recombinant virus-like particles. J. Gen. Virol. 75, 2445–2449, doi: 10.1099/0022-1317-75-9-2445 (1994). 8077946

[b22] Roden R. B. . Assessment of the serological relatedness of genital human papillomaviruses by hemagglutination inhibition. J. Virol. 70, 3298–3301 (1996). 8627814 10.1128/jvi.70.5.3298-3301.1996 PMC190197

[b23] Wang Z., Christensen N., Schiller J. T. & Dillner J. A monoclonal antibody against intact human papillomavirus type 16 capsids blocks the serological reactivity of most human sera. J. Gen. Virol. 78, 2209–2215, doi: 10.1099/0022-1317-78-9-2209 (1997). 9292008

[b24] Shank-Retzlaff M. . Correlation between Mouse Potency and *In Vitro* Relative Potency for Human Papillomavirus Type 16 Virus-Like Particles and Gardasil® Vaccine Samples. Hum. Vaccines. 1, 191–197, doi: 10.4161/hv.1.5.2126 (2005). 17012876

[b25] Roden R. . Characterization of a human papillomavirus type 16 variant-dependent neutralizing epitope. J. Virol. 71, 6247–6252 (1997). 9223527 10.1128/jvi.71.8.6247-6252.1997 PMC191893

[b26] Carter J. J., Wipf G. C., Benki S. F., Christensen N. D. & Galloway D. A. Identification of a Human Papillomavirus Type 16-Specific Epitope on the C-Terminal Arm of the Major Capsid Protein L1. J. Virol. 77, 11625–11632, doi: 10.1128/jvi.77.21.11625-11632.2003 (2003). 14557648 PMC229369

[b27] Christensen N. D. . Hybrid papillomavirus L1 molecules assemble into virus-like particles that reconstitute conformational epitopes and induce neutralizing antibodies to distinct HPV types. Virology. 291, 324–334, doi: 10.1006/viro.2001.1220 (2001). 11878901

[b28] Tyler M., Tumban E., Peabody D. S. & Chackerian B. The use of hybrid virus-like particles to enhance the immunogenicity of a broadly protective HPV vaccine. Biotechnol. Bioeng. 111, 2398–2406, doi: 10.1002/bit.25311 (2014). 24917327 PMC4410690

[b29] Hu Y. . Safety of an Escherichia coli-expressed bivalent human papillomavirus (types 16 and 18) L1 virus-like particle vaccine. Hum. Vaccin. Immunother. 10, 469–475, doi: 10.4161/hv.26846 (2013). 24161937 PMC4185883

[b30] Wu T. . Immunogenicity and safety of an E. coli-produced bivalent human papillomavirus (type 16 and 18) vaccine: A randomized controlled phase 2 clinical trial. Vaccine. 33, 3940–3946, doi: 10.1016/j.vaccine.2015.06.052 (2015). 26100924

[b31] Scherer E. M. . Characteristics of memory B cells elicited by a highly efficacious HPV vaccine in subjects with no pre-existing immunity. PLoS. Pathog. 10, e1004461, doi: 10.1371/journal.ppat.1004461 (2014). 25330199 PMC4199765

[b32] Martin A. C. R. Accessing the Kabat antibody sequence database by computer. Proteins. 25, 130–133, doi: 10.1002/(SICI)1097-0134(199605)25:1<130::AID-PROT11>3.0.CO;2-L (1996). 8727325

[b33] Joyce J. G. . The L1 Major Capsid Protein of Human Papillomavirus Type 11 Recombinant Virus-like Particles Interacts with Heparin and Cell-surface Glycosaminoglycans on Human Keratinocytes. J. Biol. Chem. 274, 5810–5822, doi: 10.1074/jbc.274.9.5810 (1999). 10026203

[b34] Richards K. F., Bienkowska-Haba M., Dasgupta J., Chen X. S. & Sapp M. Multiple Heparan Sulfate Binding Site Engagements Are Required for the Infectious Entry of Human Papillomavirus Type 16. J. Virol. 87, 11426–11437, doi: 10.1128/jvi.01721-13 (2013). 23966387 PMC3807331

[b35] Johnson K. M. . Role of Heparan Sulfate in Attachment to and Infection of the Murine Female Genital Tract by Human Papillomavirus. J. Virol. 83, 2067–2074, doi: 10.1128/jvi.02190-08 (2009). PMC2643729 19073722

[b36] Chen R., Li L. & Weng Z. ZDOCK: An initial-stage protein-docking algorithm. Proteins. 52, 80–87, doi: 10.1002/prot.10389 (2003). 12784371

[b37] Pierce B. G. . ZDOCK server: interactive docking prediction of protein-protein complexes and symmetric multimers. Bioinformatics. 30, 1771–1773, doi: 10.1093/bioinformatics/btu097 (2014). 24532726 PMC4058926

[b38] Chen R., Tong W., Mintseris J., Li L. & Weng Z. ZDOCK predictions for the CAPRI challenge. Proteins. 52, 68–73, doi: 10.1002/prot.10388 (2003). 12784369

[b39] Guan J. . Structural comparison of four different antibodies interacting with human papillomavirus 16 and mechanisms of neutralization. Virology. 483, 253–263, doi: 10.1016/j.virol.2015.04.016 (2015). 25996608 PMC4516578

[b40] White W. I. . Characterization of a Major Neutralizing Epitope on Human Papillomavirus Type 16 L1. J. Virol. 73, 4882–4889 (1999). 10233949 10.1128/jvi.73.6.4882-4889.1999 PMC112531

[b41] Zhao Q. . Disassembly and reassembly of human papillomavirus virus-like particles produces more virion-like antibody reactivity. Virol. J. 9, 52, doi: 10.1186/1743-422X-9-52 (2012). 22356831 PMC3308208

[b42] Zhao Q. . Characterization of virus-like particles in GARDASIL® by cryo transmission electron microscopy. Hum. Vaccin. Immunother. 10, 734–739, doi: 10.4161/hv.27316 (2013). 24299977 PMC4130261

[b43] Ryding J., Dahlberg L., Wallen-Öhman M. & Dillner J. Deletion of a major neutralizing epitope of human papillomavirus type 16 virus-like particles. J. Gen. Virol. 88, 792–802, doi: 10.1099/vir.0.82449-0 (2007). 17325351

[b44] Carter J. J. . Identification of human papillomavirus type 16 L1 surface loops required for neutralization by human sera. J. Virol. 80, 4664–4672, doi: 10.1128/JVI.80.10.4664-4672.2006 (2006). 16641259 PMC1472072

[b45] Zhang X. . Lessons learned from successful human vaccines: Delineating key epitopes by dissecting the capsid proteins. Hum. Vaccin. Immunother. 11, 1277–1292, doi: 10.1080/21645515.2015.1016675 (2015). 25751641 PMC4514273

[b46] Ferguson M., Wilkinson D. E., Heath A. & Matejtschuk P. The first international standard for antibodies to HPV 16. Vaccine. 29, 6520–6526, doi: 10.1016/j.vaccine.2011.07.007 (2011). 21767589

[b47] Longet S., Schiller J. T., Bobst M., Jichlinski P. & Nardelli-Haefliger D. A murine genital-challenge model is a sensitive measure of protective antibodies against human papillomavirus infection. J. Virol. 85, 13253–13259, doi: 10.1128/JVI.06093-11 (2011). 21976653 PMC3233130

[b48] Giroglou T., Florin L., Schäfer F., Streeck R. E. & Sapp M. Human Papillomavirus Infection Requires Cell Surface Heparan Sulfate. J. Virol. 75, 1565–1570, doi: 10.1128/jvi.75.3.1565-1570.2001 (2001). 11152531 PMC114064

[b49] Smith K. . Rapid generation of fully human monoclonal antibodies specific to a vaccinating antigen. Nat. protoc. 4, 372–384, doi: 10.1038/nprot.2009.3 (2009). 19247287 PMC2750034

[b50] Kondo K. . Neutralization of HPV16, 18, 31, and 58 pseudovirions with antisera induced by immunizing rabbits with synthetic peptides representing segments of the HPV16 minor capsid protein L2 surface region. Virology. 358, 266–272, doi: 10.1016/j.virol.2006.08.037 (2007). 17010405

[b51] Buck C., Pastrana D., Lowy D. & Schiller J. Efficient Intracellular Assembly of Papillomaviral Vectors. J. Virol. 78, 751–757, doi: 10.1128/JVI.78.2.751-757.2004 (2004). 14694107 PMC368835

[b52] Pastrana D. V. . Reactivity of human sera in a sensitive, high-throughput pseudovirus-based papillomavirus neutralization assay for HPV16 and HPV18. Virology. 321, 205–216, doi: 10.1016/j.virol.2003.12.027 (2004). 15051381

[b53] Buck C., Pastrana D., Lowy D. & Schiller J. Generation of HPV pseudovirions using transfection and their use in neutralization assays. Methods Mol. Med. 119, 445–62, doi: 10.1385/1-59259-982-6:445 (2005). 16350417

[b54] Wang D. . Identification of Broad-Genotype HPV L2 Neutralization Site for Pan-HPV Vaccine Development by a Cross-Neutralizing Antibody. PLoS One. 10, e0123944, doi: 10.1371/journal.pone.0123944 (2015). 25905781 PMC4408011

[b55] Chen Y. . Antigenic analysis of divergent genotypes human Enterovirus 71 viruses by a panel of neutralizing monoclonal antibodies: Current genotyping of EV71 does not reflect their antigenicity. Vaccine. 31, 425–430, doi: 10.1016/j.vaccine.2012.10.032 (2013). 23088887

[b56] Yu H. . Homology model and potential virus-capsid binding site of a putative HEV receptor Grp78. J. Mol. Model. 17, 987–995, doi: 10.1007/s00894-010-0794-5 (2011). 20628775

[b57] Pierce B. & Weng Z. ZRANK: Reranking protein docking predictions with an optimized energy function. Proteins. 67, 1078–1086, doi: 10.1002/prot.21373 (2007). 17373710

[b58] Larkin M. A. . Clustal W and Clustal X version 2.0. Bioinformatics. 23, 2947–2948, doi: 10.1093/bioinformatics/btm404 (2007). 17846036

