# Supplementary material for: A human monoclonal antibody against HPV16 recognizes an immunodominant and neutralizing epitope partially overlapping with that of H16.V5
Source: Sci Rep. 2016 Jan 11;6:19042. doi: 10.1038/srep19042 (PMC4707464; doi:10.1038/srep19042)
Supplement: Supplementary Information [file srep19042-s1.pdf]

A human monoclonal antibody against HPV16 recognizes an immunodominant and neutralizing epitope partially overlapping with that of H16.V5

Lin Xia[1+], Yangfei Xian[1+], Daning Wang[1], Yuanzhi Chen[1], Xiaofen Huang[1], Xingjian Bi[1], Hai Yu[1], Zheng Fu[1], Xinlin Liu[1], Shaowei Li[1], Zhiqiang An[1,2], Wenxin Luo[1*], Qinjian Zhao[1*], Ningshao Xia[1]

[1]State Key Laboratory of Molecular Vaccinology and Molecular Diagnostics, National Institute of Diagnostics and Vaccine Development in Infectious Diseases, School of Life Science, Xiamen University; Xiamen 361105, China.

[2]Texas Therapeutics Institute, The Brown Foundation of Molecular Medicine, University of Texas Health Science Center at Houston, Houston TX77030, USA.

[+] Lin Xia and Yangfei Xian contributed equally to this work as first author.

[*] Corresponding author: Email: wxluo@xmu.edu.cn, qinjian_zhao@xmu.edu.cn; Phone +0592 2188657; Fax +0592 2181258.

Supplementary Table S1. CDRs of the 26D1 heavy chain and light chain

**Ab chain**	**CDRs**	**Amino acid sequence**
Heavy chain	HCDR1	SSSYYWG
	HCDR2	SIHNSGNTYYNPSLKS
	HCDR3	QSDYVWRSYRRDVNFDY
Light chain	LCDR1	SGSSNIGSNTVS
	LCDR2	TNNQRPS
	LCDR3	ATWDDSLNGYV

Supplementary Table S2. Residue replacements between HPV6 and HPV16 during the preparation of hybrid VLPs

Swapping loop	Prototype sequence	Prototype sequence
	HPV6	HPV16
BC	FSIKRANKTV	FPIKKPNNNKIL
DEa	SGSGGNPGQ	ASAYAANAGV
DEb	VNVG	ECIS
DEc	VSGHPFLNKYDDV	ISGHPLLNKL
EF	KQCTNTPVQA	SPCTNVAVNP
FGa	EVGEPVPDTLII	AVGDNVPDDLYI
FGb	SGNRTSVG	SGSTANLA
FGc	IYVN	NYFP
HIa	TLCASVTT	SLCAAIST
HIb	STYTNSDY	ETTYKNTNF

Supplementary Table S3. Hydrogen bond sites between the HPV16 antigen ligand and the 26D1 hAb receptor

Epitope	Hydrogen bond	Paratope	Region
Asp128	2	Gln1<br>Tyr111X	LFR1<br>HCDR3
Asn131	1	Arg111Y	HCDR3
Ala132	1	Gln1	LFR1
Ser133	9	Ser2<br>Gly25<br>Arg80<br>Thr85<br>Ser110<br>Tyr111X	LFR1<br>LCDR1<br>LFR3<br>LCDR3<br>HCDR3
Ala134	2	Ser2<br>Arg80	LFR1<br>LFR3
Tyr135	1	Ser24	LCDR1
Asn138	3	Arg18<br>Tyr67<br>Lys72	LCDR1<br>HCDR2
Ser282	1	Pro69	HCDR2

Supplementary Figure S1.

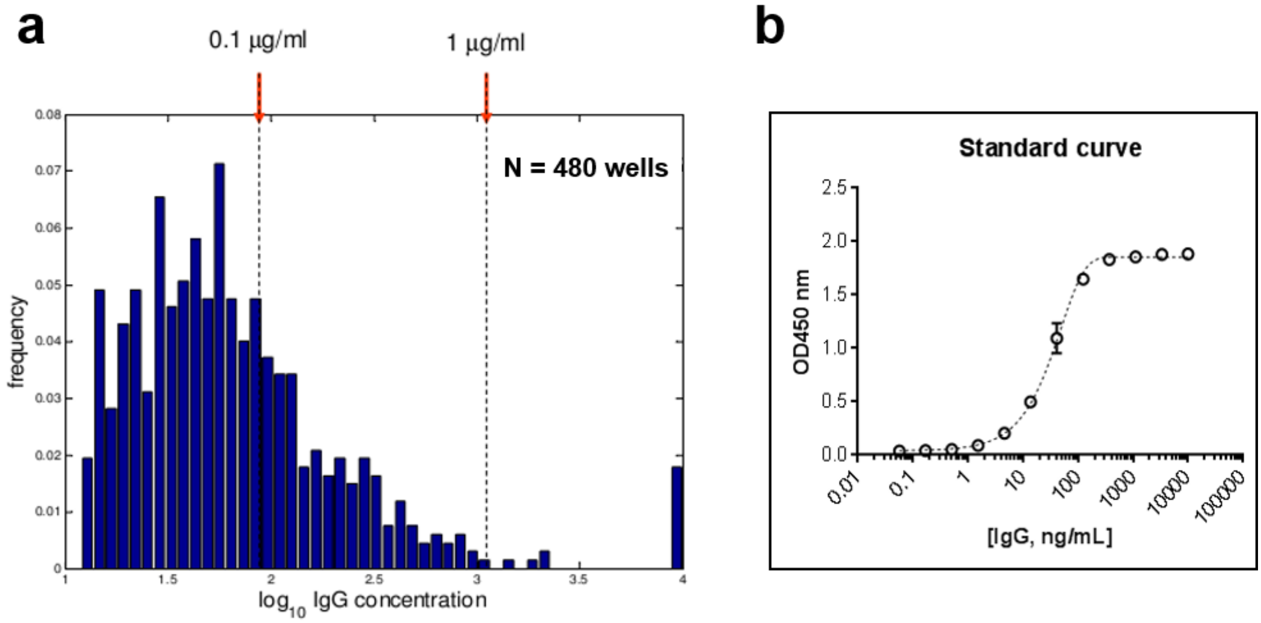

Supplementary Figure 1. IgG1 concentration distribution of memory B cell cultures. (a) IgG1 distribution of memory B cell cultures. IgG1 concentration of memory B cell culture wells were calculated using standard human IgG1 curve tested by sandwich ELISA assay as describe in method. The concentrations are plotted on X-axis with number of wells in percentage on Y-axis. 63% of IgG1 concentrations are less than 0.1 μg/ml, 34.5% of them are within the range of 0.1-1 μg/ml, only 2.5% of IgG1 concentrations are more than 1 μg/ml. Standard curve was established using purified human IgG1 whole molecule of known concentration (Pierce) with 3 fold dilutions starting at 10 μg/ml (b).

Supplementary Figure S2.

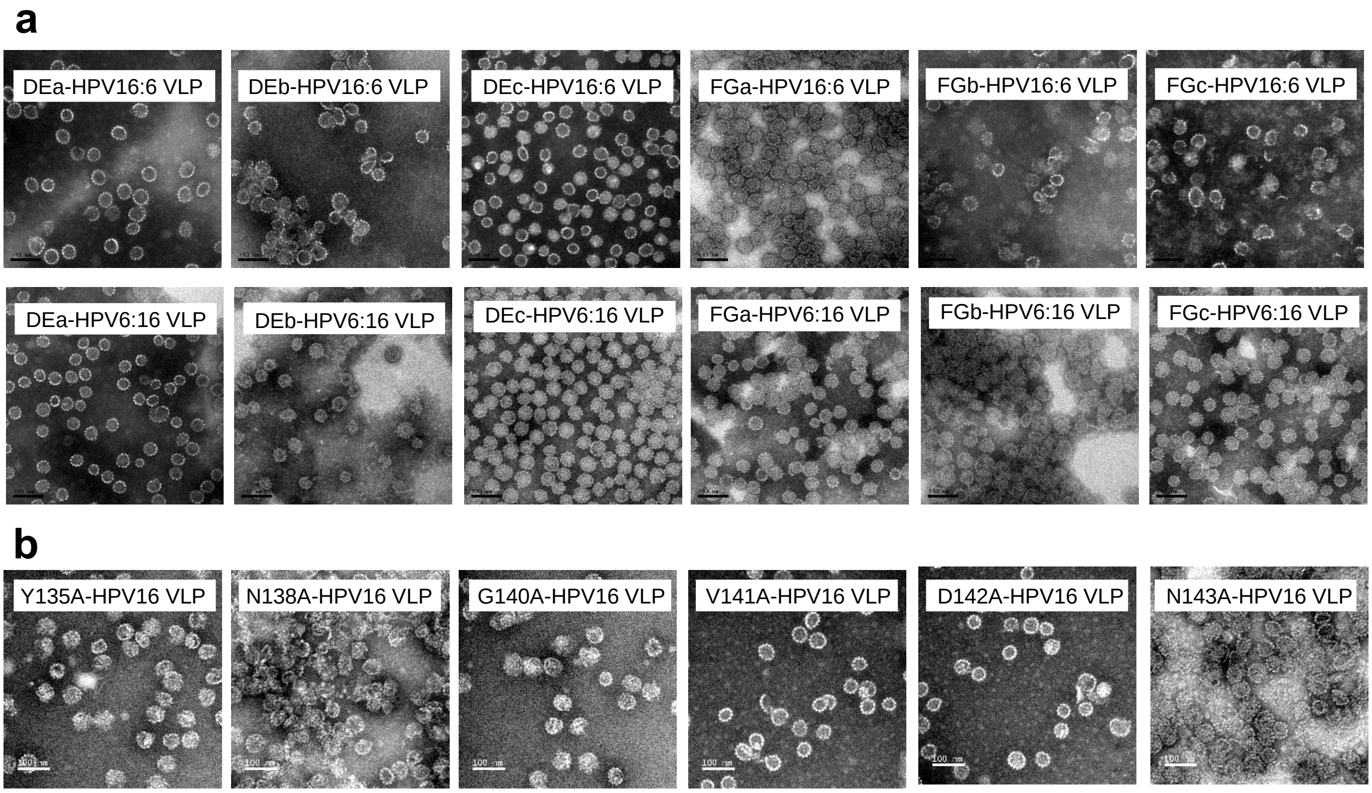

Supplementary Figure 2. TEM visualization of the recombinant proteins in the virus-like particle (VLP) form. HPV16:6 hybrid VLPs represent substituted HPV6 hypervariable loop residues for HPV16 residues on the HPV16 L1 backbone. HPV6:16 hybrid VLPs represent substituted HPV16 loop residues for HPV6 residues on the HPV6 L1 backbone. Recombinant VLP proteins were observed by TEM (Bars represent 100 nm). (a) HPV16:6 hybrid VLPs with DE and FG swapping loops are indicated (top); HPV6:16 hybrid VLPs with DE and FG swapping loops are indicated (below). Visualizations for other hybrid VLPs by TEM are not shown. (b) HPV16 point mutant VLPs with Ala substitutions on the DEa loop. Y135A-HPV16 VLP indicates that Tyr^{135} of the DEa loop was mutated to Ala on the HPV16 L1 VLP background. The other five residue substitutions are similarly indicated.
